# One nutritional symbiosis begat another: Phylogenetic evidence that the ant tribe Camponotini acquired *Blochmannia *by tending sap-feeding insects

**DOI:** 10.1186/1471-2148-9-292

**Published:** 2009-12-16

**Authors:** Jennifer J Wernegreen, Seth N Kauppinen, Seán G Brady, Philip S Ward

**Affiliations:** 1 Josephine Bay Paul Center for Comparative Molecular Biology and Evolution, Marine Biological Laboratory, Woods Hole, MA 02543, USA; 2 Department of Entomology, National Museum of Natural History, Smithsonian Institution, Washington, DC 20560, USA; 3 Department of Entomology and Center for Population Biology, University of California, Davis, CA 95616, USA; 4 Department of Integrative Biology, University of California, Berkeley, CA 94720, USA

## Abstract

**Background:**

Bacterial endosymbiosis has a recurring significance in the evolution of insects. An estimated 10-20% of insect species depend on bacterial associates for their nutrition and reproductive viability. Members of the ant tribe Camponotini, the focus of this study, possess a stable, intracellular bacterial mutualist. The bacterium, *Blochmannia*, was first discovered in *Camponotus *and has since been documented in a distinct subgenus of *Camponotus*, *Colobopsis*, and in the related genus *Polyrhachis*. However, the distribution of *Blochmannia *throughout the Camponotini remains in question. Documenting the true host range of this bacterial mutualist is an important first step toward understanding the various ecological contexts in which it has evolved, and toward identifying its closest bacterial relatives. In this study, we performed a molecular screen, based on PCR amplification of 16S rDNA, to identify bacterial associates of diverse Camponotini species.

**Results:**

Phylogenetic analyses of 16S rDNA gave four important insights: (i) *Blochmannia *occurs in a broad range of Camponotini genera including *Calomyrmex, Echinopla*, and *Opisthopsis*, and did not occur in outgroups related to this tribe (*e.g., Notostigma*). This suggests that the mutualism originated in the ancestor of the tribe Camponotini. (ii) The known bacteriocyte-associated symbionts of ants, in *Formica, Plagiolepis*, and the Camponotini, arose independently. (iii) *Blochmannia *is nestled within a diverse clade of endosymbionts of sap-feeding hemipteran insects, such as mealybugs, aphids, and psyllids. In our analyses, a group of secondary symbionts of mealybugs are the closest relatives of *Blochmannia*. (iv) *Blochmannia *has cospeciated with its known hosts, although deep divergences at the genus level remain uncertain.

**Conclusions:**

The *Blochmannia *mutualism occurs in *Calomyrmex, Echinopla*, and *Opisthopsis*, in addition to *Camponotus*, and probably originated in the ancestral lineage leading to the Camponotini. This significant expansion of its known host range implies that the mutualism is more ancient and ecologically diverse than previously documented. *Blochmannia *is most closely related to endosymbionts of sap-feeding hemipterans, which ants tend for their carbohydrate-rich honeydew. Based on phylogenetic results, we propose Camponotini might have originally acquired this bacterial mutualist through a nutritional symbiosis with other insects.

## Background

Bacteria play important roles in the success and diversification of many animal groups, and insects are especially prone to establishing long-term, mutualistic endosymbioses. An estimated 10-20% of insect species, in several taxonomic orders, depend on intracellular bacterial mutualists for their viability and reproduction [1]. These obligate associates, called 'primary' (P-) endosymbionts because they are required for host survival and fecundity, often synthesize key nutrients that are lacking in the hosts' unbalanced diet (*e.g*., plant sap or vertebrate blood) [2-4]. In all cases, the bacteria live within specialized host cells called bacteriocytes and undergo maternal transmission to developing eggs or embryos. Consistent with this stable transmission through host lineages, the phylogenies of P-endosymbionts match those of their insect hosts (*e.g*., [5-9]). This phylogenetic congruence points to host-symbiont cospeciation, which can be traced back to a single, often ancient, infection event in each host group.

Among Hymenoptera, only members of the Formicidae (ants) are known to possess bacteriocyte-associated endosymbionts. And within ants, despite the group's wide variety of symbioses with microbes [10], only three known examples of such intracellular mutualisms exist. These three cases occur in subfamily Formicinae: *Formica, Plagiolepis*, and all members of the tribe Camponotini screened to date [11-14]. Although the symbioses are similar in many respects, phylogenetic analysis of bacterial 16S rDNA implies the three bacteriocyte-associated symbioses in ants evolved independently [15].

The symbionts of *Formica *and *Plagiolepis *have not been studied in depth, but have been documented within bacteriocytes on either side of the midgut epithelium. In *Formica*, symbionts are found within queen ovarioles and in developing brood [12,13,16]. The bacteria are considered maternally transmitted, but their occurrence is erratic among *Formica *species and can vary within species depending on nutritional status [12].

Endosymbionts in the tribe Camponotini, the focus of this study, are the best-studied bacterial mutualists in ants. The bacterium, *Blochmannia*, was first discovered in *Camponotus *[14] the second largest ant genus with ~1,000 described species worldwide [17]. The bacteria occur within ant bacteriocytes, which are intercalated among midgut epithelial cells, as well as queen and worker ovaries where symbionts infect the developing oocyte and are closely integrated with host development [13,14,18,19]. Although *Blochmannia *densities apparently decline in older lab-reared workers [20], and the bacteria can be eliminated from workers treated with antibiotics [18], we have never failed to find *Blochmannia *in any *Camponotus *worker collected in the field (unpublished data), which total many hundred samples across diverse species. The retention of many nutrient biosynthetic functions within the *Blochmannia *genomes implies this symbiosis plays a nutritional role [21,22]. Likewise, experimental diet treatments indicate that *Blochmannia *provides nutrients to the host, including amino acids [23].

Among insects possessing long-term intracellular mutualists, which include several sap- and blood- feeding species, many *Camponotus *spp. stand out as true omnivores that scavenge other insects as part of their complex diet. Benefits conferred by *Blochmannia *might be critical during specific periods in host individual and colony development [23-25] (unpublished data). In addition, perhaps the symbiosis originated in an ant lineage that fed on a nutritionally unbalanced diet, such as extant arboreal *Camponotus *and *Polyrhachis *that obtain most nitrogen from plant and insect exudates [26-28]. Like many other ants, these arboreal species tend sap-feeding hemipterans, such as mealybugs, aphids, and psyllids, for their carbohydrate-rich excrement, or 'honeydew' [29].

Like other P-endosymbionts, *Blochmannia *has codiverged with its known ant hosts, reflecting an evolutionarily stable association. Several phylogenetic analyses of symbiont and host genes have demonstrated cospeciation within *Camponotus *[19,30,31]. In addition, Sameshima et al. [15] showed that *Blochmannia *occurs in *Polyrhachis *and *Colobopsis *(currently a subgenus of *Camponotus *[17] but probably a separate lineage [32]), suggesting the symbiosis originated before the divergence of these three taxa, on the order of 40 MYA.

However, due to limited sampling, the actual distribution of *Blochmannia *throughout the Camponotini is unknown. Currently, the formal description of Camponotini includes eight extant genera: *Camponotus *(including subgenus *Colobopsis*), *Polyrhachis, Calomyrmex, Echinopla*, *Opisthopsis, Forelophilus, Overbeckia*, and *Phasmomyrmex *[33]. To date, *Blochmannia *has been detected in *Camponotus *(including *Colobopsis*) and *Polyrhachis *as noted above, and Stoll et al. [34] detected a *Blochmannia*-like bacterium in association with an *Echinopla *species, based on a 367-bp region of the bacterial 16S rDNA gene. Because several Camponotini genera remain unsampled, the occurrence of *Blochmannia *within the tribe is uncertain.

For context, Dasch [12] reported that eight genera of Camponotini possess intracellular bacterial symbionts, and this number (eight) has been cited in subsequent work. However, the genera cited by Dasch have since been reclassified as subgenera of *Camponotus *(i.e., *Tanaemyrmex*, *Myrmentoma*, *Myrmosericus*, *Myrmobrachys*, *Myrmocladoecus*, *Myrmothrix*, and *Colobopsis*) [17].

In this study, our goals are (i) to better understand the actual host range of *Blochmannia*, and (ii) to identify the closest bacterial relatives and likely source of this mutualism. To this end, we screened for *Blochmannia *in a broader representation of Camponotini genera, including *Calomyrmex*, *Opisthopsis*, and *Echinopla*. We also sampled a representative of *Notostigma*, a genus formerly considered part of the Camponotini but recently placed in its own tribe [33]. To screen for symbionts, we amplified and sequenced a region of the 16S rDNA gene. Our results indicate that *Blochmannia *is more widespread within the tribe than previously documented and strongly suggest a single, ancient origin for this endosymbiosis. In addition, a close relationship with secondary endosymbionts of mealybugs suggests a potential route for the acquisition of *Blochmannia*. Specifically, the tribe Camponotini may have acquired its bacterial partner by tending honeydew-producing hemipterans.

## Results and discussion

### PCR screen for *Blochmannia*

We screened 53 representatives of the Camponotini for bacterial associates (Table 1). Specimens were identified minimally to subgenus (when applicable) and in most cases to species. The sample includes 42 *Camponotus *isolates, ten of which belong to the subgenus *Colobopsis*, as well as four *Polyrhachis*, two *Calomyrmex*, four *Opisthopsis*, and one *Echinopla *isolate. We also screened a representative of *Notostigma*, which was recently removed from the tribe [33].

**Table 1:** Ant specimens screened for bacterial associates, including 42 *Camponotus *specimens, ten of which belong to the subgenus *Colobopsis*, and representatives of *Polyrhachis, Echinopla, Calomyrmex*, and *Opisthopsis*.

Species^**1**^	ID	Subgenus^**2**^	Collector	Location	GPS	Collection code	Voucher location^**3**^
*Calomyrmex albertisi*	191		P. S. Ward	Australia (Qld)	13°43'S, 143°19'E	P. S. Ward #15325	UCDC
*Calomyrmex laevissimus*	254		P. S. Ward	Australia (Qld)	11°41'S, 142°42'E	P. S. Ward #15712	UCDC
*Echinopla australis*	253		P. S. Ward	Australia (Qld)	10°45'S, 142°31'E	P. S. Ward #15692	UCDC
*Polyrhachis decumbens*	190	*Cyrtomyrma*	P. S. Ward	Australia (Qld)	12°43'S, 143°17'E	P. S. Ward #15359	UCDC
*Polyrhachis sp*.	189	*Hagiomyrma*	P. S. Ward	Australia (Qld)	13°43'S, 143°19'E	P. S. Ward #15330	UCDC
*Polyrhachis cupreata*	252	*Hedomyrma*	P. S. Ward	Australia (Qld)	16°49'S, 145°41'E	P. S. Ward #15648	UCDC
*Polyrhachis foreli*	255	*Myrma*	P. S. Ward	Australia (Qld)	16°49'S, 145°41'E	P. S. Ward #15679	UCDC
*Opisthopsis haddoni *^a^	244		A. Andersen	Australia (NT)	12°24'S, 130°55'E		UCDC
*Opisthopsis haddoni*	256		P. S. Ward	Australia (Qld)	16°49'S, 145°41'E	P. S. Ward #15653-1	UCDC
*Opisthopsis respiciens*	192		P. S. Ward	Australia (Qld)	16°27'S, 145°22'E	P. S. Ward #15395-1	UCDC
*Opisthopsis *PG01^b^	258		P. S. Ward	Papua New Guinea	05°13'S, 145°25'E	P. S. Ward #10107	UCDC
*Camponotus quercicola*	228	*Camponotus*	S. G. Brady	USA (California)	39°14'N, 121°17'W	S. G. Brady #328	USNM
*Camponotus sp*.	241	*Myrmaphaenus*	P. S. Ward	Bolivia	13°50'S, 60°52'W	P. S. Ward #12233	UCDC
*Camponotus clarithorax*	233	*Myrmentoma*	P. S. Ward	USA (California)	32°53'N, 117°06'W	P. S. Ward #14261	UCDC
*Camponotus hyatti*	186	*Myrmentoma*	P. S. Ward	USA (California)	34°01'N, 119°48'W	P. S. Ward #14925	UCDC
*Camponotus dimorphus*	234	*Myrmobrachys*	P. S. Ward	Bolivia	17°40'S, 63°27'W	P. S. Ward #12295	UCDC
*Camponotus sp*.	261	*Myrmobrachys*	P. S. Ward	Mexico (Oaxaca)	15°40'N, 96°33'W	P. S. Ward #15578	UCDC
*Camponotus sanctaefidei*	240	*Myrmocladoecus*	P. S. Ward	Bolivia	17°27'S, 63°40'W	P. S. Ward #12423	UCDC
*Camponotus suffusus*	238	*Myrmosaulus*	P. S. Ward	Australia (SA)	32°50'S, 138°02'E	P. S. Ward #13758	UCDC
*Camponotus claviscapus*	227	*Pseudocolobopsis*	P. S. Ward	Ecuador	01°04'S, 77°37'W	P. S. Ward #11338	UCDC
*Camponotus occultus*	229	*Pseudocolobopsis*	P. S. Ward	Cuba	20°25'N, 74°34'W	P. S. Ward #14421	UCDC
*Camponotus consobrinus*	239	*Tanaemyrmex*	P. S. Ward	Australia (SA)	34°53'S, 138°43'E	P. S. Ward #13723	UCDC
*Camponotus maritimus*	185	*Tanaemyrmex*	P. S. Ward	USA (California)	37°24'N, 122°14'W	P. S. Ward #15202	UCDC
*Camponotus semitestaceus*	242	*Tanaemyrmex*	S. G. Brady	USA (California)	38°51'N, 122°24'W	S. G. Brady #123	USNM
*Camponotus sp*.	216	*Tanaemyrmex*	S.G. Brady	Brazil (São Paulo)	21°42'S, 47°28'W	S. G. Brady #344	USNM
*Camponotus sp*.	263	*Tanaemyrmex*	P.S. Ward	Mexico (Oaxaca)	16°10'N, 96°30'W	P. S. Ward #15588	UCDC
*Camponotus vicinus*	235	*Tanaemyrmex*	S. G. Brady	USA (California)	39°43'N, 122°47'W	S. G. Brady #171	USNM
*Camponotus *sp. cf. *simillimus*	199	*Tanaemyrmex*	D. M. Windsor	Panama (Chiriqui)	08°31'N, 82°12'W		UCDC
*Camponotus lownei*	230	*Thlepsepinotus*	P. S. Ward	Australia (SA)	32°50'S, 138°02'E	P. S. Ward #13741	UCDC
*Camponotus sericeiventris*	213	*Myrmepomis*	S. G. Brady	Brazil (São Paulo)	21°42'S, 47°28'W	S. G. Brady #343	USNM
*Camponotus atriceps*	203	*Myrmothrix*	D. M. Windsor	Panama (Veraguas)	07°55'N, 81°20'W		UCDC
*Camponotus atriceps*	217	*Myrmothrix*	S. G. Brady	Brazil (Maranhão)	08°37'S, 46°43'W	S. G. Brady #514	USNM
*Camponotus atriceps*	219	*Myrmothrix*	S. G. Brady	Brazil (São Paulo)	21°42'S, 47°28'W	S. G. Brady #339	USNM
*Camponotus rufipes*	220	*Myrmothrix*	S. G. Brady	Brazil (São Paulo)	21°42'S, 47°28'W	S. G. Brady #337	USNM
*Camponotus renggeri*	215	*Myrmothrix*	S. G. Brady	Brazil (São Paulo)	21°42'S, 47°28'W	S. G. Brady #340	USNM
*Camponotus renggeri*	222	*Myrmothrix*	S. G. Brady	Brazil (Maranhão)	08°37'S, 46°42'W	S. G. Brady #523	USNM
*Camponotus sp*.	221	*Myrmobrachys*	S. G. Brady	Brazil (Maranhão)	08°37'S, 46°43'W	S. G. Brady #516	USNM
*Camponotus crassus*	214	*Myrmobrachys*	S. G. Brady	Brazil (Maranhão)	08°41'S, 46°46'W	S. G. Brady #519	USNM
*Camponotus crassus*	223	*Myrmobrachys*	S. G. Brady	Brazil (São Paulo)	21°42'S, 47°28'W	S. G. Brady #338	USNM
*Camponotus sp*.	224	*Myrmobrachys*	S. G. Brady	Brazil (Maranhão)	08°37'S, 46°43'W	S. G. Brady #515	USNM
*Camponotus latangulus*	236	*Myrmocladoecus*	P. S. Ward	Ecuador	01°04'S, 77°37'W	P. S. Ward #11353	UCDC
*Camponotus sp*.	260	*Myrmamblys*	P. S. Ward	Indonesia (Nusa Tenggara Timur)	08°39'S, 120°05'E	P. S. Ward #15525	UCDC
*Camponotus nitidior*	201	*Dendromyrmex*	D. M. Windsor	Panama (Panama)	08°40'N, 79°55'W		UCDC
*Camponotus leonardi*	225	*Colobopsis*	S. G. Brady	Thailand (Nakhon Ratchasima)	14°30'N, 101°55'E	S. G. Brady #531	USNM
*Camponotus BCA-01*	188	*Colobopsis*	P. S. Ward	Mexico (Baja California Sur)	23°30'N, 110°04'W	P. S. Ward #15145	UCDC
*Camponotus conithorax*	187	*Colobopsis*	P. S. Ward	Australia (Qld)	12°46'S, 143°17'E	P. S. Ward #15340	UCDC
*Camponotus etiolatus*	264	*Colobopsis*	P. S. Ward	USA (Texas)	26°25'N, 98°15'W	P. S. Ward #15610	UCDC
*Camponotus gasseri*	243	*Colobopsis*	P. S. Ward	Australia (SA)	34°53'S, 138°43'E	P. S. Ward #13731	UCDC
*Camponotus papago*	232	*Colobopsis*	P. S. Ward	Mexico (Son)	28°58'N, 112°10'W	P. S. Ward #13458	UCDC
*Camponotus saundersi*	265	*Colobopsis*	D. W. Davidson	Brunei (Temburong)	04°32'N, 115°10'E		UCDC
*Camponotus sp*.	259	*Colobopsis*	P. S. Ward	Indonesia (Nusa Tenggara Timur)	08°31'S, 119°52'E	P. S. Ward #15521	UCDC
*Camponotus sp*.	262	*Colobopsis*	P. S. Ward	Mexico (Oaxaca)	15°57'N, 96°28'W	P. S. Ward #15584	UCDC
*Camponotus vitreus*	231	*Colobopsis*	P. S. Ward	Australia (Qld)	12°43'S, 143°17'E	P. S. Ward #15338	UCDC
non-Camponotini:							
*Notostigma carazzii*^b, c^	226		P. S. Ward	Australia (Qld)	17°26'S, 145°51'E	P. S. Ward #10006-2	UCDC

^1^In addition to several Camponotini specimens, we screened one isolate of *Notostigma*, recently removed from the Camponotini and placed in its own tribe. For nearly all samples, the 16S rDNA gene was amplified using *Blochmannia*-specific primers and the PCR product was sequenced directly. The three exceptions are as follows: ^a^Overlapping primer pairs were used to amplify bacterial 16S rDNA from *Opisthopsis haddoni *244; ^b^Cloning of PCR products was required for *Opisthopsis *PG01 and *Notostigma carazzii *226; ^c^Universal eubacterial primers SL-SR were used to amplify bacterial 16S rDNA from *Notostigma carazzii *226.

^2^Subgenera are listed when avaiable.

^3^Voucher specimens have been deposited in the Bohart Museum of Entomology, University of California, Davis (UCDC) and the National Museum of Natural History, Washington, DC (USNM).

Our screen involved PCR with primers specific to the *Blochmannia *16S rDNA gene. Direct sequencing of the resulting PCR products yielded high quality data for nearly all isolates. (The few exceptions requiring alternative methods are noted as a footnote in Table 1 and detailed in the Methods section.) The Genbank accession numbers for the 52 new sequences obtained in this study are listed in boldface in Table 2. All 16S rDNA sequences from the Camponotini isolates proved to be a close match to known *Blochmannia *strains, as detailed in the database comparisons and phylogenetic analyses below. This indicates that our specific primers amplify a wide range of *Blochmannia *lineages across the tribe, but generally did not amplify other bacteria that may associate with ants in various ways (*e.g*., on the body surface or in the gut lumen).

**Table 2:** Genbank accession numbers for 16S rDNA sequences analyzed.

Taxon^**1**^	length (bp)	**Genbank Acc. No**.^**2**^
***Calomyrmex albertisi *191**	**827**	GU226318
***Calomyrmex laevissimus *254**	**787**	GU226317
***Camponotus atriceps *203**	**825**	GU226311
***Camponotus atriceps *217**	**794**	GU226312
***Camponotus atriceps *219**	**776**	GU226313
***Camponotus BCA-01 *188**	**748**	GU226271
***Camponotus clarithorax *233**	**808**	GU226295
***Camponotus claviscapus *227**	**737**	GU226292
***Camponotus conithorax *187**	**682**	GU226279
***Camponotus consobrinus *239**	**772**	GU226300
***Camponotus crassus *214**	**766**	GU226306
***Camponotus crassus *223**	**755**	GU226305
***Camponotus etiolatus *264**	**743**	GU226274
***Camponotus gasseri *243**	**757**	GU226278
***Camponotus hyatti *186**	**819**	GU226296
***Camponotus leonardi *225**	**774**	GU226276
***Camponotus lownei *230**	**786**	GU226302
***Camponotus maritimus *185**	**785**	GU226293
***Camponotus nitidior *201**	**796**	GU226297
***Camponotus occultus *229**	**757**	GU226291
***Camponotus papago *232**	**772**	GU226272
***Camponotus quercicola *228**	**784**	GU226268
***Camponotus renggeri *215**	**786**	GU226316
***Camponotus renggeri *222**	**770**	GU226315
***Camponotus rufipes *220**	**784**	GU226314
***Camponotus sanctaefidei *240**	**743**	GU226309
***Camponotus saundersi *265**	**713**	GU226277
***Camponotus semitestaceus *242**	**785**	GU226294
***Camponotus sericeiventris *213**	**802**	GU226304
***Camponotus sp*. 216**	**803**	GU226307
***Camponotus sp*. 221**	**771**	GU226303
***Camponotus sp*. 224**	**758**	GU226298
***Camponotus sp*. 259**	**732**	GU226275
***Camponotus sp*. 260**	**766**	GU226290
***Camponotus sp*. 261**	**751**	GU226299
***Camponotus sp*. 262**	**758**	GU226273
***Camponotus sp*. 263**	**767**	GU226308
***Camponotus sp. cf. simillimus *199**	**825**	GU226310
***Camponotus suffusus *238**	**748**	GU226301
***Camponotus vicinus *235**	**805**	GU226270
***Camponotus vitreus *231ii**	**694**	GU226280
***Echinopla australis *253**	**773**	GU226319
***Opisthopsis *PG01 258 -clone 1**	**864**	GU226281
***Opisthopsis *PG01 258 -clone 2**	**864**	GU226283
***Opisthopsis haddoni *244**	**1,432**	GU226284
***Opisthopsis haddoni *256**	**768**	GU226285
***Opisthopsis respiciens *192**	**706**	GU226282
***Polyrhachis cupreata *252**	**795**	GU226289
***Polyrhachis decumbens *190**	**736**	GU226288
***Polyrhachis foreli *255**	**730**	GU226286
***Polyrhachis sp*. 189**	**904**	GU226287
		
***Notostigma carazzii *226**	**1,202**	GU226269
		
*Camponotus americanus*	1,413	41058429
*Camponotus abdominalis*	1,215	AJ245591
*Camponotus balzani*	1,509	AJ245596
*Camponotus castaneus*	1,513	AJ245594
*Camponotus chromaiodes*	1,375	41058426
*Camponotus festinatus*	1,402	AY196851
*Camponotus floridanus*	1,413	AY334381
*Camponotus herculeanus*	1,481	AJ250715
*Camponotus laevigatus*	1,373	AY334370
*Camponotus ligniperdus*	1,430	1212815
*Camponotus nipponicus*	569	AB018676
*Camponotus novaeboracensis*	1,376	41058429
*Camponotus ocreatus*	1,389	41058422
*Camponotus pennsylvanicus*	1,580	71795899
*Camponotus rufipes*	1,532	AJ245597
*Camponotus sansabeanus*	1,369	41058418
*Camponotus sayi*	1,409	41058421
*Camponotus schaefferi*	1,350	41058423
*Camponotus sericeiventris*	1,273	8250189
*Camponotus silvicola*	1,512	AJ245592
*Camponotus socius*	1,519	AJ245595
*Camponotus ulcerosus*	1,386	41058425
*Camponotus vafer*	1,410	AY334369
*Camponotus vicinus*	1,378	41058424
*Polyrhachis dives*	570	AB018678
*Polyrhachis hippomanes*	570	AB018679
*Polyrhachis lamellidens*	570	AB018680
*Polyrhachis ypsilon*	569	AB018681
		
*Aquamonas haywardensis*	1,522	AF015258
ant symbiont (*Plagiolepis manczshurica *host)	567	AB018682
ant symbiont (*Plagiolepis pigmaea *host)	567	AB018683
*Baumannia *of leafhoppers (*Oncometopia orbono *host)	1,408	57116285
*Brenneria alni*	1,501	AJ223468
*Brenneria quercina*	1,524	AJ223469
*Citrobacter freundii*	1,522	AF025365
*Erwinia amylovora*	1,464	AJ746201
*Erwinia carotovora*	1,458	U80198
*Escherichia coli*	1,542	U00096
*Ewingella americana*	1,392	EU678360
*Formica fusca symbiont*	568	AB018684
*Hafnia alvei*	1,479	M59155
*Hafnia sp*. 270	1,508	AM403659
*Hafnia sp*. NJ-71	1,522	AM419021
*Hamiltonella defensa *(T-type 2° symbiont of aphids)	1,447	AF293616
*Klebsiella pneumoniae*	1,489	AJ233420
*Leminorella grimontii*	1,482	AJ233421
lousefly 2° symbiont (*Pseudolynchia canariensis *host)	1,525	DQ115535
mealybug 2° symbiont (*Amonostherium lichtensioides *host)	1,504	AF476100
mealybug 2° symbiont (*Antonia crawii *host)	1,465	6978941
mealybug 2° symbiont (*Australicoccus grevilleae *host)	1,555	21717581
mealybug 2° symbiont (*Cyphonococcus alpinus *host)	1,559	21717584
mealybug 2° symbiont (*Dysmicoccus brevipes *host)	1,517	AF476103
mealybug 2° symbiont (*Melanococcus albizziae *host)	1,528	AF476106
mealybug 2° symbiont (*Paracoccus nothofagicola *host)	1,570	21717591
mealybug 2° symbiont (*Planococcus citri *host)	1,560	21717589
*Pasteurella multocida*	1,432	AY507110
*Pectobacterium carotovorum*	1,544	BX950851
*Photorhabdus luminescens*	1,545	BX571861
*Plesiomonas shigelloides*	1,499	X60418
*Pseudomonas aeruginosa*	1,536	AE004883
psyllid 2° symbiont (*Aphalaroida inermis *host)	1,513	8575696
psyllid 2° symbiont (*Blastopsylla occidentalis *host)	1,511	AF263558
*Rahnella aquatilis*	1,520	X79939
*Rahnella sp. NJ-8*	1,506	AM419020
*Regiella insecticola *(U-type 2° symbiont of aphids)	1,387	AF293623
*Salmonella typhimurium*	1,544	AE008857
*Serratia marcescens*	1,505	AJ233431
*Shigella flexneri*	1,541	AE015280
*Sodalis glossinidius*, 2° symbiont of tsetse flies - GM-SG1	1,507	AY861701
*Sodalis glossinidius*, 2° symbiont of tsetse flies - str. 'morsitans'	1,511	AP008232
tephritid fruit fly symbiont (*Noeeta pupillata *host)	1,313	EF469633
*Vibrio cholerae*	1,535	AE004096
weevil symbiont (*Sitophilus oryzae*) host	1,512	AF548137
weevil symbiont (*Sitophilus oryzae*) host, strain SFr	1,461	AF005235
weevil symbiont (*Sitophilus zeamais *host)	1,509	AF548142
*Xenorhabdus nematophilus*	1,497	X82251
*Yersinia pestis*	1,585	AE013985
*Yersinia sp*.	1,506	AJ011333

^1^Bacterial sequences from Camponotini are labeled by the ant host species.

^2^The 52 new sequences obtained in this study are listed in boldface.

The 16S rDNA amplified from an outgroup related to this tribe (*Notostigma*) did not match *Blochmannia*. In addition, previous work has screened other members of the subfamily Formicinae for bacterial symbionts and failed to detect *Blochmannia*. The bacterial symbiont in *Formica *and *Plagiolepis *is not *Blochmannia*, as we have verified in our phylogenetic analysis here. Moreover, we were unable to amplify *Blochmannia *from a specimen of *Oecophylla smaradina *(unpublished data).

### Database comparisons

We compared new sequences to Genbank and to the Ribosomal Database Project (RDP, release 10.5) [35]. These searches are based on sequence similarity and provide a very rough idea of taxonomic affiliation. Most new bacterial 16S rDNA sequences from the Camponotini most closely matched published *Blochmannia *in *both *databases. (Please see additional file 1 for a list of top matches.) These include isolates of *Calomyrmex *and *Opisthopsis*, where *Blochmannia *has not been detected before, and *Echinopla*, where a short, *Blochmannia*-like sequence was noted once previously [34]. For an additional four sequences, *Blochmannia *was the top match in one of the two databases, and nearly the top in the other database. Four members of the *Colobopsis *subgenus most closely match other members of the Enterobacteriaceae. Nonetheless, subsequent phylogenetic analysis (below) groups these *Colobopsis *associates with *Blochmannia *with very high confidence, illustrating the approximate nature of the similarity-based database searches. The bacterial associate of *Notostigma carazzii *did not match *Blochmannia *in either database.

### Phylogenetic results

#### Gamma-Proteobacteria analysis: Camponotini isolates group with known *Blochmannia *strains

**Figure 1:**
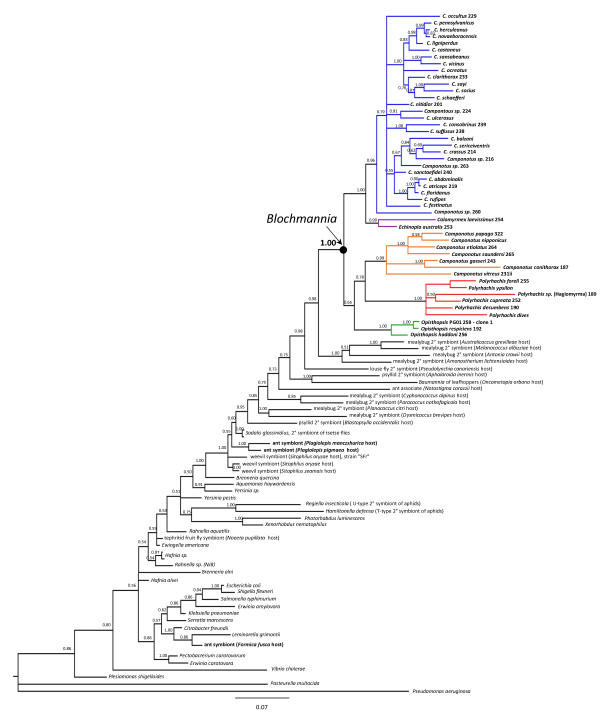
**Relationships among diverse gamma-Proteobacteria, estimated from a region of the 16S rDNA gene**. Within *Blochmannia*, taxa are labeled by the ant host from which the bacterial gene was amplified. These and other ant symbionts are noted in boldface. The phylogeny was estimated using Bayesian methods. The topology shown reflects the majority-rule consensus of post-burnin trees, and posterior probabilities are given at nodes. The results support the monophyly of known *Blochmannia *isolates and newly sampled Camponotini associates (posterior probability of 1.00), demonstrating for first time that *Calomyrmex, Echinopla*, and *Opisthopsis *possess bacterial associates that are members of the same clade as known *Blochmannia *strains. *Blochmannia *occurs within a large, diverse, and strictly-endosymbiotic group that includes a wide range of insect endosymbionts. *Plagiolepis *and *Formica *endosymbionts do not group with *Blochmannia*, showing independent origins of intracellular endosymbioses within ants.

To test for the monophyly of presumed *Blochmannia *isolates and to position this group within the gamma-Proteobacteria, we estimated the phylogeny of 16S rDNA using Bayesian and maximum likelihood (ML) approaches. Taxa included candidates for the closest relatives of *Blochmannia*, based on matches in databases such as Genbank, the RDP [35], and ARB [36]. Bayesian posterior probabilities were higher than ML bootstrap values, as expected based on previous studies [37]. As a consequence, the Bayesian consensus tree (Figure 1) showed higher resolution than did the ML bootstrap consensus tree. (The ML tree of the gamma-Proteobacteria dataset is presented as additional file 2.) Both methods support the monophyly of known *Blochmannia *isolates and newly sampled Camponotini associates (Bayesian posterior probability of 1.00 and bootstrap support of 78%). The phylogenies also illustrate that *Plagiolepis *and *Formica *endosymbionts represent independent lineages within the gamma-Proteobacteria, as suggested in an earlier study based on a smaller taxon sample [15]. Interestingly, the *Notostigma *isolate is closely related to *Blochmannia *but clearly falls outside of that clade. This absence of *Blochmannia *is consistent with the removal of *Notostigma *from the Camponotini and assignment to its own tribe [32,33]. Whether the *Notostigma *isolate represents a symbiosis or casual bacterial associate is uncertain.

These results demonstrate for the first time that *Calomyrmex, Echinopla*, and *Opisthopsis *possess bacterial associates that are part of the same clade as known *Blochmannia *strains, consistent with a single origin of endosymbiosis in the Camponotini. Although additional Camponotini taxa remain to be screened from more rarely collected genera, we predict that *Blochmannia *will be pervasive throughout the tribe. The three Camponotini genera not included in our study (*Forelophilus, Overbeckia*, and *Phasmomyrmex*) together comprise only six living species and represent a comparatively small component of the overall diversity within the tribe. Indeed, current taxonomy suggests that *Forelophilus *and *Overbeckia *may be synonymous with *Camponotus *[33].

This discovery of *Blochmannia *in diverse Camponotini genera helps to refine the questions we ask about the evolution of this association. For example, it remains mysterious why the mutualism became established in this group, but apparently not in other ant clades. What is distinct about Camponotini? This remains an open question. Studying the full range of hosts will highlight conspicuous exceptions, where the life history or physiology of ants may impact the symbiosis. For example, while *Blochmannia *may be involved in claustral founding of new colonies [38], *Polyrhachis *shows instances of semi-claustral founding, the only known example in a formicine ant [39]. In addition, in a tribe in which the metapleural gland is often missing [33], any role of symbionts in pathogen defense may be particularly important (D. E. Wheeler and J. F. A. Traniello, pers. comm.). This gland is a structure unique to ants whose external secretions have been hypothesized to serve antiseptic functions, although other functions have been proposed that involve chemical defense, recognition odor, and territorial marking (U. Mueller, pers. comm.). The metapleural gland is present in workers from the vast majority of ant genera, including *Calomyrmex, Opisthopsis *and *Echinopla*, but is absent from *Polyrhachis *and most species of *Camponotus*. It is known to be present in the workers of at least 13 *Camponotus *species including *C. gigas *[40], *C. thadeus *[41], *C. sericeus *[42], and ten species of *Camponotus *(*Myrmonesites*) from Madagascar (B. Fisher and U. Mueller, pers. comm.). These exceptions to the rule offer natural experiments to explore the influence of host biology on symbiont persistence and functions.

Our phylogenetic analysis of gamma-Proteobacteria position *Blochmannia *within a large, diverse group of insect endosymbionts (Figure 1). This group includes many secondary symbionts of sap-feeding mealybugs, psyllids, and aphids. In our analyses, a group of secondary symbionts of mealybugs are the closest relatives of *Blochmannia*. This group represents one of at least four distinct acquisitions of gamma-Proteobacterial symbionts by mealybugs [43]. The most recent common ancestor (MRCA) of *Blochmannia *is reconstructed with very high likelihood to be endosymbiotic, with a proportional likelihood of 0.9999 under both Mk1 model and AsymmMk model (expressed as the proportion of the total probability of 1.0). Similarly, the ancestral node representing the MRCA of *Blochmannia *and the four closely-related mealybug endosymbionts is inferred to have been endosymbiotic (proportional likelihood 0.9999 under both models).

Camponotini is one of the major ant groups that commonly tend mealybugs, aphids, and other hemipterans for their carbohydrate-rich honeydew [29,44] and may have acquired *Blochmannia *through this route. Ants and hemipterans have a long history of association, with fossil evidence for this relationship occurring in Baltic (~44 MYA) and Dominican (15-20 MYA) amber deposits [45,46]. Ants often have been observed to scavenge or kill the hemipterans that they tend and transport the bodies to their nests, presumably to help feed their colonies [47-50]. Repeated consumption by ant larvae, the only stage that can ingest particulate matter, may have selected for sequestration of a hemipteran symbiont. Feeding interactions have been implicated in the horizontal transmission of other endosymbiotic bacteria in insects including *Wolbachia *[51] and *Rickettsia *[52] although definitive experimental evidence for the successful establishment of an endosymbiont via this pathway remains lacking [52].

#### *Blochmannia*-focused analysis

**Figure 2:**
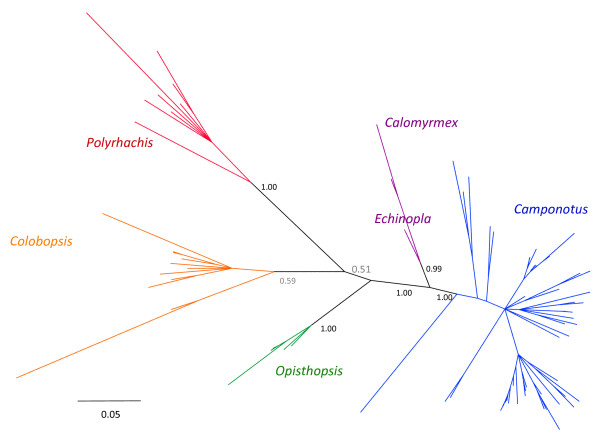
***Blochmannia *phylogeny estimated from a region of the 16S rDNA gene, analyzed without outgroups**. The analysis includes 50 new bacterial sequences from Camponotini and 28 published *Blochmannia *sequences. Taxon groups are labeled by the ant host from which the bacterial gene was amplified. The phylogeny was estimated using Bayesian methods, and the topology shown reflects the majority-rule consensus of post-burnin trees. Both this unrooted and rooted (Figure 3) phylogenies resolved four major lineages within *Blochmannia: Polyrhachis*, *Colobopsis*, *Opisthopsis*, and a fourth group composed of *Camponotus*, *Calomyrmex*, and *Echinopla*. In this unrooted tree, only the posterior probabilities of major nodes are marked. Relationships within major groups resemble those in the rooted tree (Figure 3). Taxon names were removed for clarity, and we refer the reader to the rooted tree for these data.

**Figure 3:**
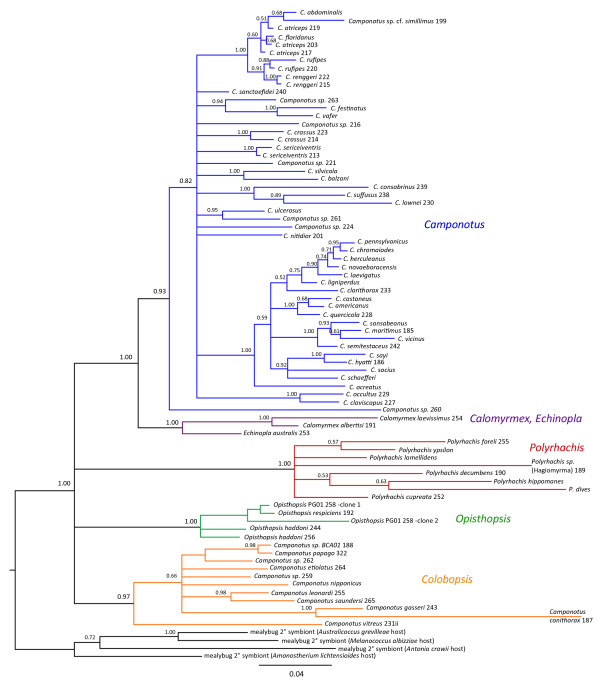
***Blochmannia *phylogeny estimated from a region of the 16S rDNA gene, analyzed with outgroups**. The analysis includes 50 new bacterial sequences from Camponotini, 28 published *Blochmannia *sequences, and four mealybug endosymbionts that we found to be the closest relatives to *Blochmannia*. Taxa are labeled by the ant host from which the bacterial gene was amplified. The phylogeny was estimated using Bayesian methods, and the topology shown reflects the majority-rule consensus of post-burnin trees. Posterior probabilities of all nodes are marked. Taxon names of new samples are followed by a sample ID number, whereas published *Blochmannia *sequences are not. Like the unrooted tree (Figure 2), this rooted phylogeny resolved four major lineages within *Blochmannia: Polyrhachis*, *Colobopsis*, *Opisthopsis*, and a fourth group comprised of *Camponotus*, *Calomyrmex*, and *Echinopla*.

We performed a more focused phylogenetic analysis that included new bacterial sequences obtained here, as well as 28 published *Blochmannia *sequences. We analyzed the data with and without mealybug endosymbionts as outgroups. Using Bayesian approaches, the unrooted (Figure 2) and rooted (Figure 3) phylogenies resolved four subgroups within *Blochmannia *that match genus-level distinctions among their ant hosts: *Polyrhachis*, *Camponotus *subgenus *Colobopsis *(called *Colobopsis *here, for brevity), *Opisthopsis*, and a fourth group composed of *Camponotus*, *Calomyrmex*, and *Echinopla*. In rooted Bayesian trees, each subgroup was resolved with high confidence (posterior probabilities of 0.97-1.00). The ML analyses gave similar results but with lower confidence. Unrooted and rooted ML trees of *Blochmannia *are presented in additional files 3 and 4, respectively.

**Figure 4:**
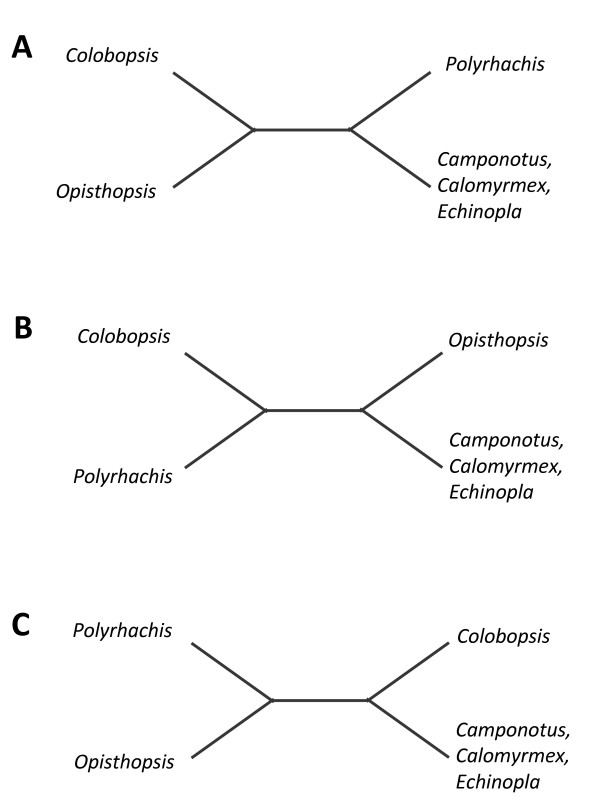
**Schematic trees reflecting the possible relationships among the four well-supported *Blochmannia *lineages detected here**. For four lineages, three possible unrooted trees exist (A, B, C), each with five possible root positions, or 15 trees total. The Shimodaira-Hasegawa test indicated that we cannot distinguish among the three possible unrooted or 15 possible rooted topologies (see text and Tables 3, 4).

In an analysis of ant relationships, Brady et al. [32] found strong support for the grouping of *Polyrhachis *with *Camponotus *+ *Calomyrmex*, the relationship reflected in schematic tree A (Figure 4). While the rooting of the Camponotini was uncertain, it may occur along the *Opisthopsis *lineage [32]. By contrast, our 16S rDNA dataset does not distinguish relationships among major subgroups within *Blochmannia*. For example, we considered the three possible unrooted trees for these four lineages (trees A-C; Figure 4). The Shimodaira-Hasegawa (SH) test indicated the data cannot distinguish among the three possible unrooted topologies (Table 3). To test whether our dataset can resolve the root position, we also used the SH test to evaluate the relative support for 15 trees that represent the alternative rootings of trees A-C. The data do not reject any of the 15 alternative trees, and thus cannot reject any hypotheses for relationships among major *Blochmannia *lineages (Table 4). Additional data from rDNA genes or other *Blochmannia *loci will be necessary to resolve ancient divergences within this mutualism.

**Table 3:** Results of the Shimodaira-Hasegawa (SH) test indicate the data cannot reject any of the three alternative unrooted *Blochmannia *phylogenies.

Constraint tree^**1**^	-ln L	Diff -ln L	p-value^**2**^
A	8785.83	0.52	0.67
B	8785.31	(best)	
C	8785.97	0.66	0.74

^1^The four major *Blochmannia *lineages may be related in three possible ways, reflected in three unrooted trees A-C (see Figure 4).

^2^Non-significant results indicate that the likelihood score does not differ significantly from that of the "best" tree (topology B).

**Table 4:** Results of the Shimodaira-Hasegawa (SH) test indicate the data cannot reject any of the 15 alternative rooted *Blochmannia *phylogenies.

Constraint tree^**1**^	Root Position^**2**^	-ln L	Diff -ln L	p-value^**3**^
A	Camp+Cal+Echin	9895.98	3.63	0.86
A	Colob	9892.35	(best)	
A	Mid	9900.48	8.14	0.80
A	Opis	9899.04	6.69	0.82
A	Poly	9899.24	6.90	0.84
B	Camp+Cal+Echin	9895.73	3.38	0.88
B	Colob	9896.81	4.46	0.85
B	Mid	9894.59	2.24	0.97
B	Opis	9895.78	3.44	0.95
B	Poly	9893.82	1.47	0.90
C	Camp+Cal+Echin	9895.18	2.84	0.91
C	Colob	9894.88	2.53	0.94
C	Mid	9896.98	4.64	0.88
C	Opis	9906.04	13.69	0.66
C	Poly	9898.01	5.66	0.81

^1^Each of the three unrooted trees (A-C, see Figure 4) has five possible root positions, for 15 possible rooted trees.

^2^The root position indicates the lineage to which outgroup taxa (mealybug endosymbionts) were attached, with "mid" indicating the internal branch.

^3^Non-significant results indicate that the likelihood score does not differ significantly from that of the "best" tree (topology A-Colob).

#### Agreement of bacterial and ant relationships

Agreement of the *Blochmannia *and host phylogeny is expected, given that several previous studies demonstrate host-symbiont cospeciation in this mutualism [15,19,30,31]. While a formal cospeciation analysis is beyond the scope of this study, the *Blochmannia *phylogeny suggests host-symbiont congruence at taxonomic levels previously unexamined. At the tribe level, we detected *Blochmannia *only in association with Camponotini, and not, for example, in *Notostigma*. At the genus level, the major groups within *Blochmannia *match distinctions among the ant hosts. In a comprehensive phylogenetic analysis of ants, Brady et al. [32] confirmed that *Camponotus *is a polyphyletic assemblage. *Colobopsis*, although still formally considered a subgenus of *Camponotus *[17], constitutes a distinct group that is separated from *Camponotus *by intervening genera. Here, we found an identical pattern in the 16S rDNA of associated *Blochmannia*. That is, *Blochmannia *from ants in the subgenus *Colobopsis *formed a group that is distinct from other *Camponotus*. In addition, the close relationship of bacteria from *Calomyrmex *and *Camponotus *matches the affiliation of these ant genera [32,53].

At shallower taxonomic levels, many well-resolved species-level relationships among *Blochmannia *agree with known or predicted host relationships. Congruence has already been documented in previous cospeciation studies of many species included here [15,19,30,31]. In addition, our new data illustrate further examples of recently-diverged *Blochmannia *strains from ant hosts that are close relatives [54]. Examples include *C. quercicola *and *C. castaneus, C. rufipes *and *C. floridanus*, *C. hyatti *and *C. sayi*, *C. balzani *and *C. silvicola*, and the group containing *C. sansabeanus, C. maritimus, C. semitestaceus *and *C. vicinus*. Although *Camponotus *subgenera are often polyphyletic and not always good predictors of host relationships [31,54], notably the *Blochmannia *of *C. claviscapus *and *C. occultus*, both members of the subgenus *Pseudocolobopsis*, are sister taxa. Furthermore, *Blochmannia *sampled from the same *Camponotus *species but from different geographic areas are always closely related, usually as a strongly supported monophyletic unit.

### Caveats: Strengths and limitations of molecular data

The molecular approach presented here has clear advantages. This approach let us screen for symbionts using one or few host specimens, infer relationships among the bacteria detected, and place newly-discovered isolates within a broader phylogenetic context. However, based on DNA sequence data alone, it is difficult to make strong conclusions about the type of association between *Blochmannia *and the additional genera sampled here. For instance, it is impossible to say for certain that the bacteria form stable, obligate, intracellular relationships in each host genus - i.e., that the type of mutualism well-characterized in *Camponotus *also occurs in *Opisthopsis*, *Calomyrmex*, and *Echinopla*. A complete characterization of *Blochmannia *in these genera is beyond the scope of the current study, but ideally will include additional representatives of each genus to test for symbiont persistence and stability, and will be coupled with ultrastructural work to document the location of bacteria within host tissues or cells. Such characterizations will be a fruitful area for future research.

Despite these caveats, the demonstrated specificity of *Blochmannia *makes its detection in additional host genera compelling. When a symbiont group is already well described, the presence of that symbiont is frequently based on molecular data alone, even when it is detected in new hosts. In addition, our results support the hypothesis that *Blochmannia *is monophyletic and evolved more than 40 MYA from an ancient, diverse clade consisting of intracellular endosymbionts. This pattern lends further support to the view that newly-discovered *Blochmannia *lineages within the Camponotini also live within cells. Moreover, the general agreement of host and symbiont phylogenies bolsters the view that these bacterial associates form meaningful, stable associations with their hosts.

## Conclusions

Our molecular screen and phylogenetic analysis of ant-associated bacteria revealed four important results.

(i) We demonstrated for the first time that *Calomyrmex *and *Opisthopsis *possess bacterial associates that are members of the same clade as known *Blochmannia *strains. Furthermore, we confirmed a similar finding for *Echinopla*. These results significantly expand the range of known hosts of this symbiont and suggest the mutualism is more ancient and diverse than previously documented. Consistent with the recent removal of *Notostigma *from the Camponotini [33], we were unable to detect *Blochmannia *in a specimen of this genus. Although additional Camponotini genera remain to be screened (*Forelophilus, Overbeckia*, and *Phasmomyrmex*), we predict that *Blochmannia *will be pervasive throughout the Camponotini.

(ii) The three known bacteriocyte-associated symbionts in ants evolved independently in *Formica, Plagiolepis*, and the Camponotini. The three symbionts constitute distinct lineages within the gamma-Proteobacteria.

(iii) *Blochmannia *is positioned within a diverse, strictly endosymbiotic bacterial group and is reconstructed with very high likelihood to have originated from an endosymbiotic ancestor. This larger bacterial group includes endosymbionts of mealybugs, psyllids, lice, weevils, *Plagiolepis*, and tsetse flies. Our analysis suggests that a group of secondary symbionts of mealybugs constitute the closest relatives to *Blochmannia*, based on available sequence data, and suggest a possible origin for the ant mutualism. Unlike primary symbionts such as *Blochmannia*, secondary symbionts form dynamic associations and are known to transfer among distinct insect species and, occasionally, among insect superfamilies [55]. Camponotini might have acquired the bacteria by tending mealybugs or other sap-feeding hemipterans for their carbohydrate-rich honeydew.

(iv) We found that the phylogeny of *Blochmannia *agrees with known relationships among Camponotini hosts at taxonomic levels previously unexamined. We detected four robust groups within *Blochmannia*: isolates from *Polyrhachis*, *Colobopsis, Opisthopsis*, and a fourth group composed of *Camponotus *(excluding *Colobopsis*), *Calomyrmex*, and *Echinopla*. Our data further support *Colobopsis *as a lineage distinct from remaining *Camponotus*.

Exciting areas for future research include testing the prediction that *Blochmannia *are pervasive throughout the Camponotini, by characterizing microbial associates of unsampled genera and by documenting the tissue- and cellular location of bacteria detected. Additional phylogenetic analysis, ideally based on expanded molecular and morphological datasets, will shed light on the deep divergences among Camponotini genera and whether hosts and symbionts codiverged in the earliest stages of this ancient mutualism.

## Methods

### Molecular methods

For each ant sample, genomic DNA was prepared from whole ants or from only the gaster (QIAGEN DNeasy kit). In nearly all cases, single worker ants were used. Rarely, two or more ant individuals from the same colony were combined for a given DNA prep, particularly when ants were small. For samples that gave very low yields of DNA, we amplified the DNA using GenomiPhi (GE Life sciences). As a positive control for DNA quality and quantity, we PCR-amplified a 1.3-kb region of cytochrome oxidase I and II (*COI/II*). Nearly all samples gave a visible *COI/II *product. These PCR products generally were not sequenced for this study, but rather served as an indicator of high-quality DNA.

We used primers designed to match *Blochmannia *16S rDNA to screen for this endosymbiont across ant specimens. These specific primers (Bloch16S-462F 5-AAACCCTGATGCAGCTATACCGTGTGTG-3', and Bloch16S-1299R 5'-CCATTGTAGCACGTTTGTAGCCCTACTCA-3') produce a PCR product of ~840 bp [31]. 16S rDNA PCR reactions were repeated in large-scale (50 uL) format and the products purified (Promega Wizard PCR purification kit). PCR products were sequenced directly in both directions on an ABI 3730×l automated sequencer using Big Dye v3.0 (Applied Biosystems). This approach gave high quality DNA sequences for nearly all samples. All sequences were assembled and edited using PHRED, PHRAP and CONSED. DNA assemblies were checked by eye and any ambiguous base calls were re-sequenced or changed to N. Most sequences obtained were ~750-800 bp, slightly shorter than the PCR product itself.

Three isolates required the use of alternative primer pairs and/or cloning, in order to obtain high quality data. These few exceptions are noted in the footnote in Table 1 and detailed here. First, for *Opisthopsis *PG01, the PCR product generated from *Blochmannia-*specific primers was cloned (Invitrogen TOPO TA kit). Six clones were selected for sequencing with M13F and M13R primers and showed considerable variation. Two sequences had a highest BLASTn hits to known *Blochmannia *isolates, and were included in the phylogenetic analyses presented here; the remaining four sequences showed closest BLASTn hits to bacteria from soils. While the latter four sequences might represent meaningful bacterial associates, we favor the conservative interpretation that they reflect environmental contaminants. Notably, we found significant variation between the two *Blochmannia *clones. Because two *Opisthopsis *PG01 individuals were pooled for the gDNA preparation (one worker and one pupa), it is possible that this reflects standing genetic variation between host individuals, a subject for future investigation.

Two samples (*Notostigma carazzii *226 and *Opisthopsis haddoni *244) did not give detectable PCR products with the *Blochmannia*-specific primers. For *Notostigma carazzii *226, we used the eubacterial 16S primers SL and SR [31] to amplify a 1,202 bp fragment that was subsequently cloned and sequenced with M13F and M13R primers. Among the eight clones sequenced, only minor variation was observed, likely reflecting cloning artifacts. The single sequence used in our phylogenetic analyses was selected for its length and high quality. Finally, for *Opisthopsis haddoni *244, we generated two overlapping PCR products using the primers 16S_10F (5'-AGTTTGATCATGGCTCAGATTG-3') + 23S_480R (5'-CACGGTACTGGTTCACTATCGGTC-3'), and 16S_777F (5'-AGCAAACAGGATTAGATACCC-3') + SR; these were sequenced directly to generate a 1,432-bp fragment.

Among the bacterial 16S rDNA sequences from Camponotini, all matched known *Blochmannia*, based on database comparisons and/or phylogeny reconstructions (see Results). Only three specimens, *Camponotus latangulus *236, *Campontous sp*. 241, and *Camponotus dimorphus *234, failed to work in our molecular screen. These isolates did not generate PCR for host *COI/II*, our positive control for DNA quality. In addition, a *C. vitreus *worker that we initially sampled apparently possessed two bacterial associates: *Blochmannia*, as well as an isolate that matched the soil bacterium *Chromohalobacter*. To test whether the latter was a persistent bacterial associate or contaminant, we screened a second *C. vitreus *worker collected from the same colony. The second worker (*C. vitreus *231ii) possessed only *Blochmannia *and is included in the phylogenetic analyses here.

### Database comparisons

New bacterial 16S rDNA sequences were compared to all sequences in two comprehensive databases. First, we identified closest matches in the Ribosomal Database Project (RDP, release 10.5; http://rdp.cme.msu.edu/) using the Sequence Match utility [35]. This allowed us to compare our new sequences to 671,510 high quality Bacterial SSU sequences. In addition, we used BLASTn for a more general comparison of our new sequences to all sequences in Genbank. These searches are based on DNA sequence similarity and provide a very rough prediction of taxonomic affiliation. The advantage of this approach is the ability to search numerous 16S rDNA sequences very rapidly. The disadvantage is that the similarity results are not always good indicators of phylogenetic relationships. Nonetheless, these broad comparisons helped us to identify the closest non-*Blochmannia *outgroups to include in phylogenetic analysis.

### Phylogenetic analysis

#### Taxon selection

Taxa were selected according to the goals of two phylogenetic analyses. *(i) Gamma-Proteobacteria analysis*. First, we explored the broad placement of *Blochmannia *16S rDNA sequences, including new sequences obtained here, within the gamma-Proteobacteria. This analysis (a) tested whether newly discovered bacterial associates of Camponotini form a monophyletic group that includes known *Blochmannia*, (b) identified the closest relative to *Blochmannia*, and (c) tested whether *Plagiolepis *and *Formica *endosymbionts group with *Blochmannia *or, alternatively, represent distinct symbiont acquisitions in ants. The full, 99-taxon dataset for this gamma-Proteobacteria analysis included representatives of new and published *Blochmannia *sequences, endosymbionts of other insects, non-endosymbiotic Enterobacteriaceae, and divergent outgroup taxa. To identify the immediate relatives of *Blochmannia*, we included its closest matches in databases such as Genbank, the Ribosomal Database Project [35], and ARB [36]. We deliberately excluded the exceptionally AT-rich primary endosymbionts (e.g., *Wigglesworthia, Buchnera, Carsonella*) to reduce biases resulting from extreme base compositions, especially the artefactual grouping of AT-rich sequences [56,57]. In addition, initial analyses showed their phylogenetic positions were unstable across the tree.

*(ii) Blochmannia*-*focused analysis*. Second, we explored relationships among *Blochmannia *in a targeted analysis of 78 bacterial isolates from Camponotini, including the previously unsampled host genera, *Calomyrmex, Echinopla*, and *Opisthopsis*. We analyzed *Blochmannia *sequences with and without sequences of four mealybug endosymbionts that proved to be the closest outgroups in the gamma-Proteobacteria analysis above.

#### Sequence alignment

16S rDNA sequences were aligned using SILVA INcremental Aligner, or SINA http://www.arb-silva.de/aligner/[36]. The alignment was examined carefully and any regions considered ambiguous were excluded from analysis. This 16S rDNA alignment, with ambiguous regions marked, is provided in nexus format as additional file 5. After excluding ambiguous regions, alignment lengths were 1,510 sites (599 of which were variable) for the gamma-Proteobacteria analysis and 1,547 sites (429 variable) for the *Blochmannia*-focused analysis. At 700-800 bp, many *Blochmannia *sequences were considerably shorter than the full alignment length and thus included stretches of missing data. However, we chose to analyze a longer region to retain as much information as possible.

#### Phylogenetic reconstruction

For both the gamma-Proteobacteria and *Blochmannia*-focused analyses, datasets were analyzed using Modeltest 3.7 to determine the appropriate model of sequence evolution [58]. Based on the hierarchical likelihood ratio test (hLRT) results, the most appropriate model for both datasets is the General Time Reversible model with invariant sites and rate variation among sites (GTR+I+G).

We performed phylogenetic analysis with Bayesian and maximum likelihood (ML) methods. *Bayesian analysis*. Using the parallel version of MrBayes v3.1.2, we implemented a GTR model with unequal base frequencies, portion of invariant sites estimated from the data, rate variation among sites according to the gamma distribution, and noninformative priors. We simultaneously performed two independent runs, each with four incrementally heated Markov Chain Monte Carlo (MCMC) chains starting from a random tree. Analyses were run for 10 million generations with trees sampled every 100 generations. Stationarity of log likelihood (-lnL) was confirmed by plotting the -lnL scores versus the number of generations. Discarding the first 90,000 trees provided a very conservative 90% burn-in, as -lnL was stationary well before this point. Posterior probabilities were determined by constructing a 50% majority-rule tree of the 10,000 trees sampled after the burn-in. To assess whether Bayesian runs had adequate convergence and mixing for a given data set, we confirmed that the convergence diagnostic PSRF (pscale reduction factor) approached or reached one in all cases, indicating convergence of the two independent runs. In addition, we used Compare and other functions available in AWTY (Are We There Yet; http://ceb.scs.fsu.edu/awty) to confirm convergence [59].

##### Maximum likelihood analysis

We performed ML analysis using GARLI Version 0.96 (Beta) https://www.nescent.org/wg_garli/. We used the default GTR model, in light of Modeltest results supporting a relatively complex model. Base frequencies and the portion of invariant sites were estimated from the data, and rates among sites were allowed to vary according to a discrete gamma distribution (4 categories). The runs were terminated when no topology with an lnL increase of 0.01 or greater had been found in 20,000 generations. We used these parameters to find the trees with the highest likelihood score and to perform bootstrap analyses of 100 replications.

We used likelihood tests to compare alternative hypotheses regarding: (i) possible relationships among the four deep *Blochmannia *lineages, represented by three unrooted trees, and (ii) the 15 various attachments of outgroups to the unrooted *Blochmannia *trees (three unrooted trees × five possible root attachments = 15). We first estimated the most likely tree (using GARLI as described above) under the appropriate topological constraints (e.g., the unrooted trees A-C shown in Figure 3, and the 15 possible attachments of outgroups to those unrooted trees). We then compared these ML trees using the Shimodaira-Hasegawa (SH) test [60] as implemented by PAUP version 4.0b10 for Unix [61]. These SH tests involved optimizing each data set across the topologies being compared, using the GTR model described above, then evaluating whether the -lnL of a given dataset differed significantly between the "best" versus alternative topologies. The SH test was performed with all taxa in the *Blochmannia*-focused analysis.

##### Ancestral state reconstruction

We used ancestral state reconstruction to infer the history of endosymbiosis in this group, using the Bayesian majority consensus tree with branch lengths and employing the maximum likelihood methods provided by Mesquite v.2.6 [62]. Taxa were coded as either endosymbiotic or non-endosymbiotic. We employed two different models of character evolution, the Mk1 model which uses a single parameter for the rate of change and the AsymmMk model which uses two parameters and thus allows for a bias in gains versus losses.

## Authors' contributions

JJW performed the DNA sequence alignment, phylogenetic analysis, and database comparisons. SNK, as a research assistant at the MBL, performed the molecular biology lab work and assisted with data analysis. SGB and PSW provided ant specimens, identifications, and phylogenetic and myrmecological expertise. SGB performed ancestral character state reconstruction. All authors contributed to the design of the study, commented on the manuscript, and approved the final version.

## Authors' information

JJW explores the evolution of insect endosymbionts, studying the effects of lifestyle transitions (e.g., from free-living to host-dependent) on bacterial genomes and the specific roles of symbionts within their insect hosts. SNK is currently a graduate student at the University of California, Berkeley, where he studies the role of symbiotic associations in the generation and maintenance of tropical plant diversity. SGB is a Curator of Hymenoptera and Research Entomologist at the National Museum of Natural History whose research focuses on systematics, molecular evolution, coevolution, and social evolution, especially in ants and bees. PSW is an ant systematist whose research is concerned with delimiting species, inferring phylogenetic relationships, and seeking a better understanding of ant evolution.

## Supplementary Material

Additional file 1 **Database matches for 52 new bacterial 16S rDNA sequences obtained in this study**. The vast majority of sequences most closely matched a published *Blochmannia *16S rDNA sequence in NCBI (compared using BLASTn) and/or the Ribosomal Database Project (RDP; compared using SeqMatch). Click here for file

Additional file 2 **Maximum likelihood phylogeny of gamma-Proteobacteria, estimated from a region of the 16S rDNA gene**. Within *Blochmannia*, taxa are labeled by the ant host from which the bacterial gene was amplified. The topology reflects the majority-rule consensus tree of 100 bootstrap replicates. Bootstrap values ≥70% are marked. (All unmarked nodes have bootstrap values of 50%-69%.) Click here for file

Additional file 3 **Maximum likelihood phylogeny of *Blochmannia*, estimated without outgroups**. Taxa are labeled by the ant host from which the bacterial gene was amplified. The topology reflects the majority-rule consensus tree of 100 bootstrap replicates. In this unrooted tree, only bootstrap values of deep nodes are marked. Otherwise, relationships resemble those in the fully-labeled rooted tree (see additional file 4). Click here for file

Additional file 4 **Maximum likelihood phylogeny of *Blochmannia*, estimated with outgroups**. Taxa are labeled by the ant host from which the bacterial gene was amplified. The topology reflects the majority-rule consensus tree of 100 bootstrap replicates. In this rooted tree, bootstrap values ≥70% are marked. (All unmarked nodes have bootstrap values of 50%-69%.) Outgroup taxa are four mealybug endosymbionts that we found to be the closest relatives to *Blochmannia*. Support for the monophyly of subgenus *Colobopsis *is 55%. Click here for file

Additional file 5 **MacClade file (nexus format) of the 16S rDNA sequence alignment analyzed here**. Ambiguous (excluded) regions in the alignment are marked as character sets. Sequences used in phylogenetic analyses are marked as distinct taxon sets. Click here for file
