# Supplementary material for: One nutritional symbiosis begat another: Phylogenetic evidence that the ant tribe Camponotini acquired Blochmannia by tending sap-feeding insects
Source: BMC Evol Biol. 2009 Dec 16;9:292. doi: 10.1186/1471-2148-9-292 (PMC2810300; doi:10.1186/1471-2148-9-292)
Supplement: Additional file 1 — Database matches for 52 new bacterial 16S rDNA sequences obtained in this study. The vast majority of sequences most closely matched a published Blochmannia 16S rDNA sequence in NCBI (compared using BLASTn) and/or the Ribosomal Database Project (RDP; compared using SeqMatch). [file 1471-2148-9-292-S1.pdf]

Additional file 1. Database matches for 52 new bacterial 16S rDNA sequences obtained in this study.

Ant specimen	Query length (bp)	Best BLASTn hit	GB number of best BLASTn hit	E-value	*Blochmannia* rank[1]	Best match in RDP	GB number of best RDP match	RDP similarity score	*Blochmannia* rank[1]
Calomyrmex albertisi 191	827	*Blochmannia - C. ulcerosus*	AY334375	0	1	*Blochmannia - C. festinatus*	AY196851	0.965	1
Calomyrmex laevissimus 254	787	*Blochmannia - C. ulcerosus*	AY334375	0	1	*Blochmannia - C. festinatus*	AY196851	0.956	1
Camponotus clarithorax 233	808	*Blochmannia - C. ligniperdus*	X92551	0	1	*Blochmannia - C. ligniperdus*	X92551	0.988	1
Camponotus claviscapus 227	739	*Blochmannia - C. vafer*	AY334369	0	1	*Blochmannia - C. vafer*	AY334369	0.978	1
Camponotus conithorax 187	682	*Blochmannia - C. ulcerosus*	AY334375	0	1	*Blochmannia - C. ulcerosus*	AY334375	0.946	1
Camponotus consobrinus 239	772	*Blochmannia - C. ulcerosus*	AY334375	0	1	*Blochmannia - C. vafer*	AY334369	0.965	1
Camponotus hyatti 186	819	*Blochmannia - C. sayi*	AY334371	0	1	*Blochmannia - C. sayi*	AY334371	0.989	1
Camponotus maritimus 185	785	*Blochmannia - C. sansabeanus*	AY334368	0	1	*Blochmannia - C. sansabeanus*	AY334368	0.989	1
Camponotus vicinus 235	805	*Blochmannia - C. vicinus*	AY334374	0	1	*Blochmannia - C. vicinus*	AY334374	0.985	1
Camponotus occultus 229	757	*Blochmannia - C. vafer*	AY334369	0	1	*Blochmannia - C. vafer*	AY334369	0.974	1
Camponotus quercicola 228	784	*Blochmannia - C. castaneus*	AY334377	0	1	*Blochmannia - C. castaneus*	AY334377	0.993	1
Camponotus sanctaefidei 240	747	*Blochmannia - C. floridanus*	BX248583	0	1	*Blochmannia - C. floridanus*	AY334381	0.980	1
Camponotus semitestaceus 242	785	*Blochmannia - C. sansabeanus*	AY334368	0	1	*Blochmannia - C. sansabeanus*	AY334368	0.988	1
Camponotus sp. cf. simillimus. 199	825	*Blochmannia - C. abdominalis*	AJ245591	0	1	*Blochmannia - C. abdominalis*	AJ245591	0.988	1
Camponotus nitidior 201	796	*Blochmannia - C. castaneus*	AY334377	0	1	*Blochmannia - C. festinatus*	AY196851	0.978	1
Camponotus atriceps 203	825	*Blochmannia - C. floridanus*	BX248583	0	1	*Blochmannia - C. floridanus*	AY334381	0.999	1
Camponotus atriceps 217	794	*Blochmannia - C. floridanus*	BX248583	0	1	*Blochmannia - C. floridanus*	AY334381	0.999	1
Camponotus atriceps 219	776	*Blochmannia - C. abdominalis*	AJ245591	0	1	*Blochmannia - C. floridanus*	AY334381	0.997	1
Camponotus rufipes 220	784	*Blochmannia - C. rufipes*	X92552	0	1	*Blochmannia - C. rufipes*	X92552	0.999	1
Camponotus sericeiventris 213	802	*Blochmannia - C. sericeiventris*	AJ245593	0	1	*Blochmannia - C. sericeiventris*	AJ245593	0.997	1
Camponotus crassus 214	766	*Blochmannia - C. ulcerosus*	AY334375	0	1	*Blochmannia - C. abdominalis*	AJ245591	0.979	1
Camponotus crassus 223	755	*Blochmannia - C. ulcerosus*	AY334375	0	1	*Blochmannia - C. vafer*	AY334369	0.983	1
Camponotus renggeri 215	787	*Blochmannia - C. rufipes*	X92552	0	1	*Blochmannia - C. rufipes*	X92552	0.995	1
Camponotus renggeri 222	770	*Blochmannia - C. rufipes*	X92552	0	1	*Blochmannia - C. rufipes*	X92552	0.994	1
Camponotus sp. 216	803	*Blochmannia - C. vafer*	AY334369	0	1	*Blochmannia - C. vafer*	AY334369	0.974	1
Camponotus sp. 221	771	*Blochmannia - C. ulcerosus*	AY334375	0	1	*Blochmannia - C. fellah*	EF422835	0.980	1
Camponotus sp. 224	758	*Blochmannia - C. ulcerosus*	AY334375	0	1	*Blochmannia - C. ulcerosus*	AY334375	0.981	1
Camponotus lownei 230	786	*Blochmannia - C. ulcerosus*	AY334375	0	1	*Blochmannia - C. herculeanus*	X92550	0.967	1
Camponotus sp. 260	766	*Blochmannia - C. vafer*	AY334369	0	1	*Blochmannia - C. vafer*	AY334369	0.969	1
Camponotus sp. 261	751	*Blochmannia - C. ulcerosus*	AY334375	0	1	*Blochmannia - C. ulcerosus*	AY334375	0.978	1
Camponotus sp. 263	767	*Blochmannia - C. vafer*	AY334369	0	1	*Blochmannia - C. abdominalis*	AJ245591	0.984	1
Camponotus suffusus 238	748	*Blochmannia - C. balzani*	AJ245596	0	1	*Blochmannia - C. sansabeanus*	AY334368	0.962	1
Camponotus vitreus 231ii	694	*Blochmannia - C. sericeiventris*	AJ245593	0	1	*Blochmannia - Polyrhachis foreli*	AY336986	0.948	1
Echinopla australis 253	773	*Blochmannia - C. abdominalis*	AJ245591	0	1	*Blochmannia - C. festinatus*	AY196851	0.965	1
Opisthopsis haddoni 244	1432	*Blochmannia - C. ulcerosus*	AY334375	0	1	*Blochmannia - C. vitiosus*	AB018675	0.955	1
Opisthopsis haddoni 256	768	*Blochmannia - C. ulcerosus*	AY334375	0	1	*Blochmannia - C. festinatus*	AY196851	0.958	1
Opisthopsis respiciens 192	706	*Blochmannia - C. ulcerosus*	AY334375	0	1	*Blochmannia - C. festinatus*	AY196851	0.960	1
Polyrhachis cupreata 252	795	*Blochmannia - Polyrhachis foreli*	AY336986	0	1	*Blochmannia - Polyrhachis foreli*	AY336986	0.955	1
Polyrhachis foreli 255	730	*Blochmannia - Polyrhachis foreli*	AY336986	0	1	*Blochmannia - Polyrhachis foreli*	AY336986	0.996	1
Polyrhachis decumbens 190	736	*Blochmannia - Polyrhachis foreli*	AY336986	0	1	*Blochmannia - Polyrhachis foreli*	AY336986	0.962	1
Polyrhachis sp. 189	904	*Blochmannia - Polyrhachis foreli*	AY336986	0	1	*Blochmannia - Polyrhachis lamellidens*	AB018680	0.944	1
Camponotus leonardi 225	775	*Blochmannia - C. floridanus*	BX248583	0	1	uncultured bacterium	DQ124751	0.950	4
Camponotus gasseri 243	757	*Blochmannia - C. ulcerosus*	AY334375	0	1	2° symbiont of *Planococcus citri* (mealybug)	AF322016	0.944	>20
Camponotus etiolatus 264	743	*Blochmannia - C. vafer*	AY334369	0	1	*Plesiomonas shigelloides*	DQ822740	0.954	>20
Camponotus sp. 259	732	2° symbiont of *Planococcus citri* (mealybug)	AF476107	0	3	*Blochmannia - C. festinatus*	AY196851	0.957	1
Camponotus BCA-01 188	748	2° symbiont of *Planococcus citri* (mealybug)	AF476107	0	6	*Enterobacteriaceae bacterium LA20*	DQ822739	0.953	>20

Camponotus sp. 262	758	2° symbiont of *Planococcus citri* (mealybug)	AF476107	0	13	*Enterobacteriaceae bacterium LA20*	DQ822739	0.958	>20
Camponotus saundersi 265	713	Uncultured bacterium clone PB1_aai26f03	EU460325	0	5	*Rahnella sp. Nj-65*	AJ842247	0.954	>20
Camponotus papago 232	772	Uncultured bacterium from *Tetropium* (beetle)	AM946408	0	6	*Enterobacteriaceae bacterium LA20*	DQ822739	0.953	>20
Notostigma carazzi 226	1202	*Sodalis glossinidius str. 'morsitans'*	AP008232	0	44	uncultured bacterium	EU136818	0.949	>20
Opisthopsis PG01 258 -clone 1	864	*Blochmannia - C. festinatus*	AY196851	0	1	*Blochmannia - C. festinatus*	AY196851	0.965	1
Opisthopsis PG01 258 -clone 2	864	*Blochmannia - C. ulcerosus*	AY334375	0	1	*Pantoea sp. LUP*	EF474461	0.977	3

[1] "*Blochmannia* rank" lists the order that a *Blochmannia* sequence first appeared in the list of top hits. When this value is ">20" for the RDP comparisons, no *Blochmannia* sequences appeared in the top 20 matches reported. The vast majority (45/50) of sequences most closely matched a published *Blochmannia* 16S rDNA sequence in NCBI (compared using BLASTn) and/or the Ribosomal Database Project (RDP; compared using SeqMatch). These numbers exclude the two *Opisthopsis* PG01 clones, which were were selected based on their match to *Blochmannia* (see Methods) and are listed in grey font at the end of the table.
