# Supplementary figures and images for: One nutritional symbiosis begat another: Phylogenetic evidence that the ant tribe Camponotini acquired Blochmannia by tending sap-feeding insects
Source: BMC Evol Biol. 2009 Dec 16;9:292. doi: 10.1186/1471-2148-9-292 (PMC2810300; doi:10.1186/1471-2148-9-292)

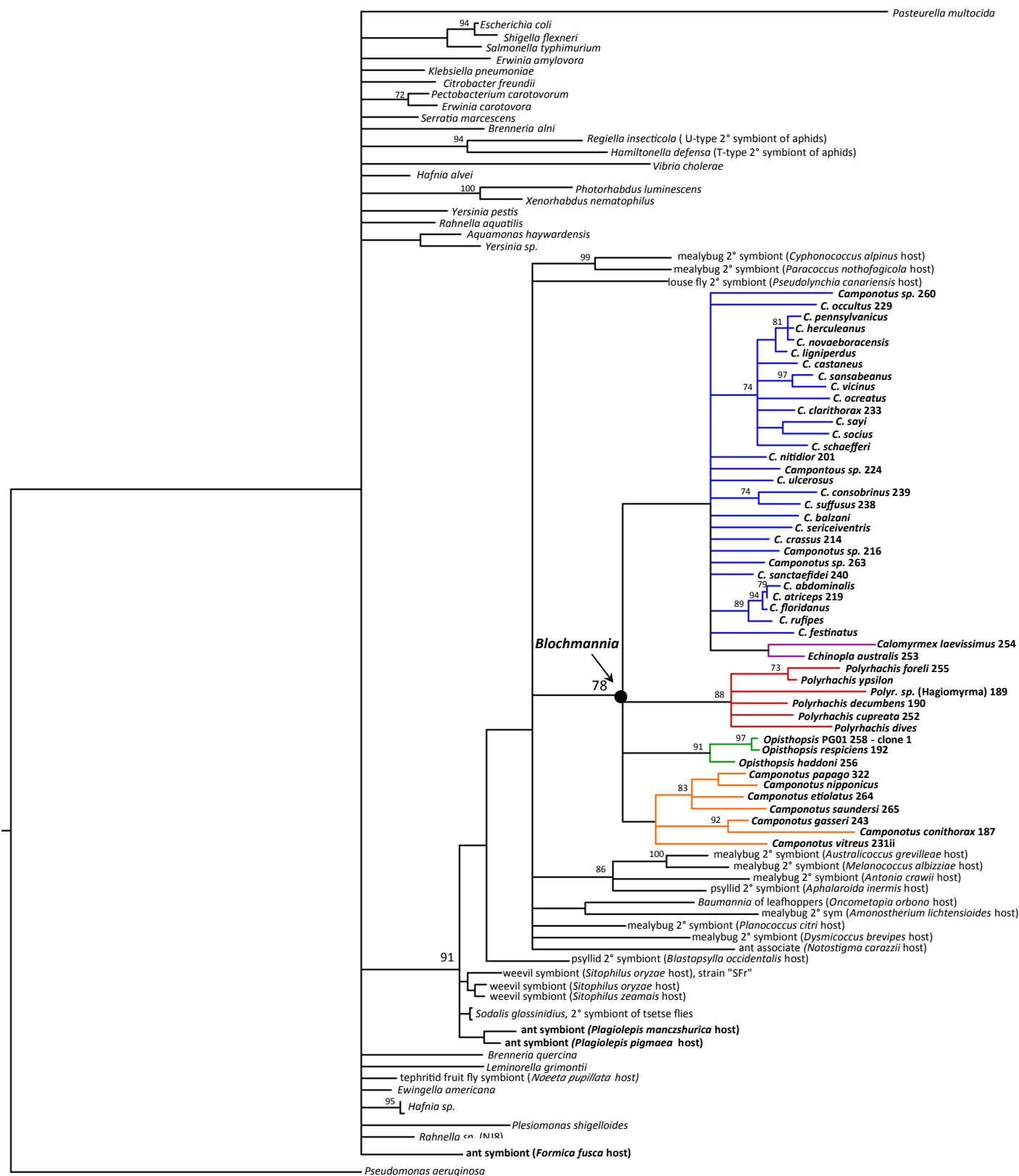

0.1

Supplement: Additional file 2 — Maximum likelihood phylogeny of gamma-Proteobacteria, estimated from a region of the 16S rDNA gene. Within Blochmannia, taxa are labeled by the ant host from which the bacterial gene was amplified. The topology reflects the majority-rule consensus tree of 100 bootstrap replicates. Bootstrap values ≥70% are marked. (All unmarked nodes have bootstrap values of 50%-69%.) [file 1471-2148-9-292-S2.pdf]

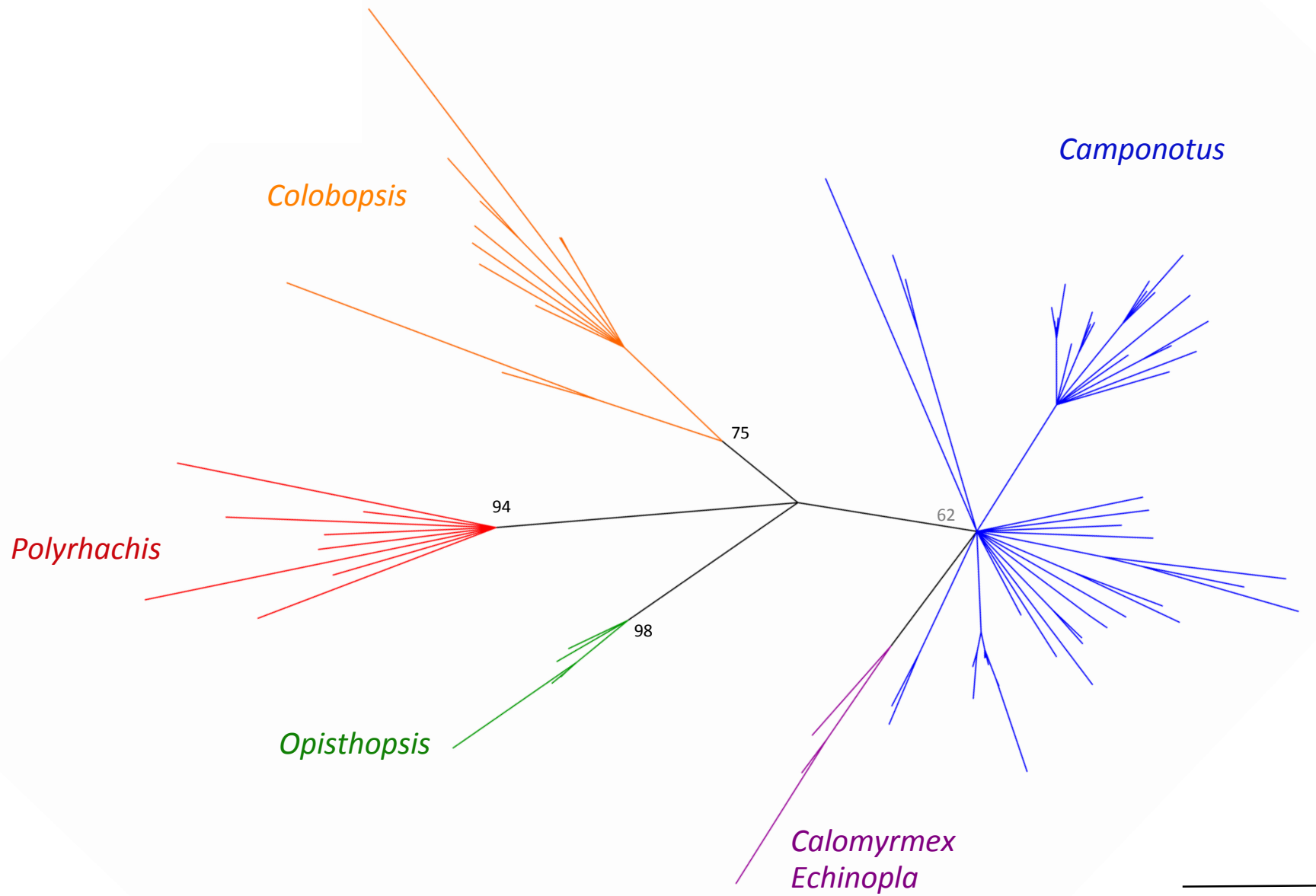

Supplement: Additional file 3 — Maximum likelihood phylogeny of Blochmannia, estimated without outgroups. Taxa are labeled by the ant host from which the bacterial gene was amplified. The topology reflects the majority-rule consensus tree of 100 bootstrap replicates. In this unrooted tree, only bootstrap values of deep nodes are marked. Otherwise, relationships resemble those in the fully-labeled rooted tree (see additional file 4). [file 1471-2148-9-292-S3.pdf]

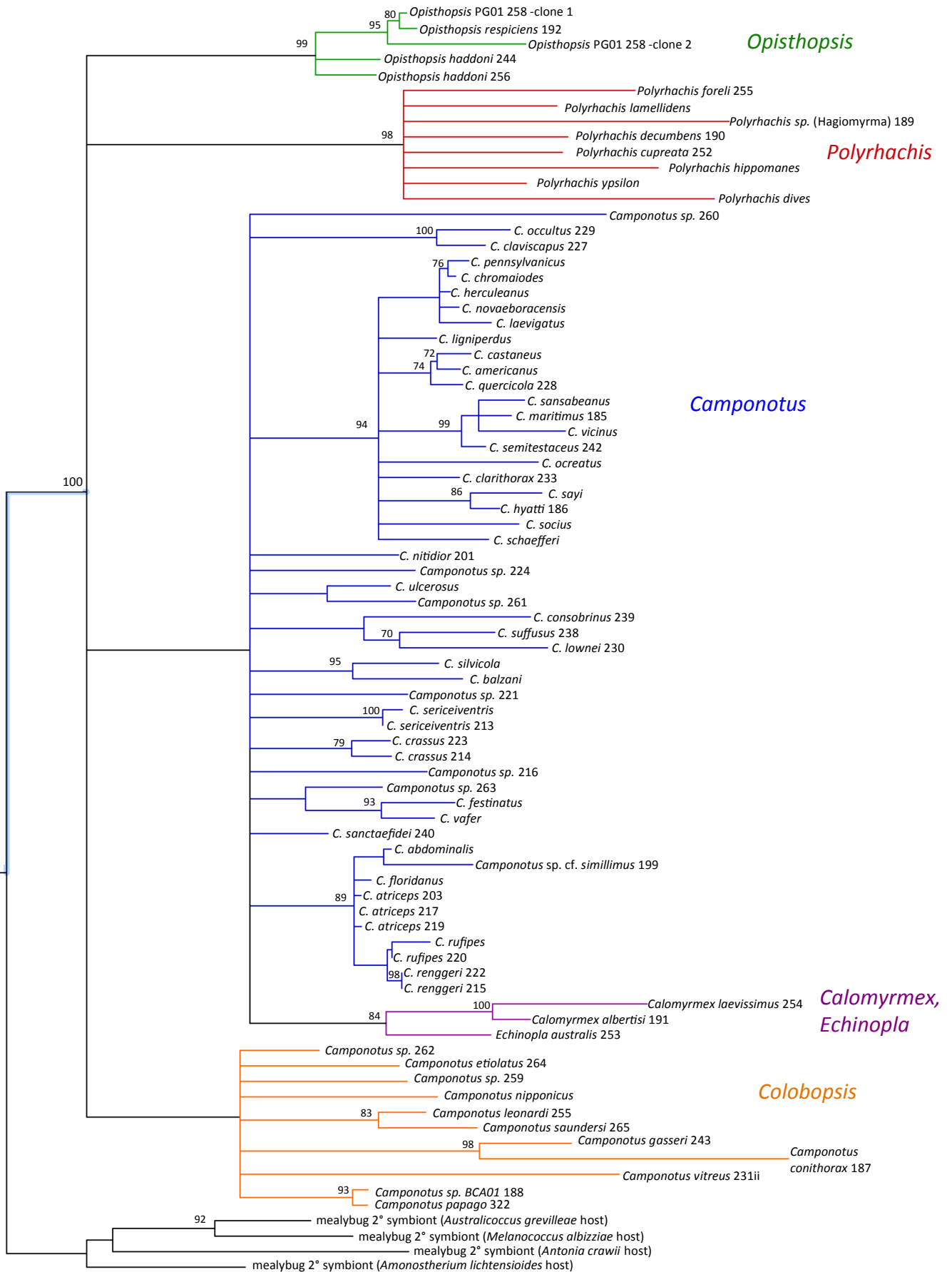

Supplement: Additional file 4 — Maximum likelihood phylogeny of Blochmannia, estimated with outgroups. Taxa are labeled by the ant host from which the bacterial gene was amplified. The topology reflects the majority-rule consensus tree of 100 bootstrap replicates. In this rooted tree, bootstrap values ≥70% are marked. (All unmarked nodes have bootstrap values of 50%-69%.) Outgroup taxa are four mealybug endosymbionts that we found to be the closest relatives to Blochmannia. Support for the monophyly of subgenus Colobopsis is 55%. [file 1471-2148-9-292-S4.pdf]
